# REQUIREMENTS FOR POLICY MEASURES THAT FAVOR BETTER WORKING CONDITIONS FOR THE LONG-TERM CARE WORKFORCE IN EUROPE

**DOI:** 10.1093/geroni/igae098.1950

**Published:** 2024-12-31

**Authors:** Montserrat Gea-Sánchez, Jose Mateos

**Affiliations:** University of Lleida, Lleida, Catalonia, Spain; University of Lleida, Lleida, Catalonia, Spain

## Abstract

There is a need to improve key working conditions, such as low wages, high rates of part-time employment, and insufficient autonomy and support for workers. In addition, the pandemic has highlighted the need for better coordination between the long-term care sector and the wider health system, and recruitment and training policies must be accompanied by strategies to improve management within the sector. A narrative review of the main measures carried out by several national European administrations to improve working conditions in long-term care was conducted. Documentation was retrieved from a scientific literature review, grey literature and key informants (n = 9). The results of the review suggest that the long-term care sector suffers from structural deficiencies that were accentuated during the COVID-19 pandemic. Among these deficiencies the following were highlighted: the lack of professionals in long-term care mainly due to poor working conditions and gaps in training and role definition, especially for nurses. Measures to reverse this situation, such as the integration of new technologies or the replacement of nurses by other professionals who have acquired some of their competencies have been implemented in some European countries. These measures, in addition to being insufficient, may lead to a decline in the quality of care. Persistent problems of high turnover and low retention pose a threat to both accessibility and quality of services.

